# Recent Progress in Curcumin Extraction, Synthesis, and Applications: A Comprehensive Review

**DOI:** 10.3390/foods15020354

**Published:** 2026-01-18

**Authors:** Qirui Meng, Feng Xiao, Dahai Jiang, Wenxuan Jiang, Wenze Lin, Huiliang Gan, Tong Ye, Jianchun Jiang, Liming Lu

**Affiliations:** 1 Academy of Advanced Carbon Conversion Technology, Huaqiao University, Xiamen 361021, China; 2 Fujian Provincial Key Laboratory of Biomass Low-Carbon Conversion, Huaqiao University, Xiamen 361021, China; 3 College of Chemical Engineering, Huaqiao University, Xiamen 361021, China; 4 The Institute of Chemical Industry of Forest Products, Chinese Academy of Forestry, Nanjing 210042, China

**Keywords:** curcumin, extraction technologies, microbial biosynthesis, bioavailability, industrial applications

## Abstract

Curcumin, a natural polyphenol derived from *Curcuma longa* L., exhibits diverse biological activities including anti-inflammatory, anticancer, and antioxidant effects, making it a versatile candidate for food, feed, pharmaceutical, and cosmetic applications. However, its industrial application is hindered by low bioavailability, poor water solubility, and high production costs. This review comprehensively summarizes the latest advances in curcumin’s physicochemical properties, production routes (phytoextraction, chemical synthesis, and microbial biosynthesis), and wide applications. Compared with existing reviews, this work emphasizes quantitative benchmarking of production methods (yield, productivity, and environmental metrics), critical evaluation of application feasibility including regulatory hurdles and clinical evidence, and actionable future directions for industrial scalability. We systematically analyze the advantages, limitations, economic and environmental trade-offs of each production route, and highlight recent innovations in bioavailability enhancement and metabolic engineering. This review aims to provide a holistic theoretical and technical framework for accelerating curcumin’s sustainable development and commercialization in high-value products.

## 1. Introduction

**Figure 1:**
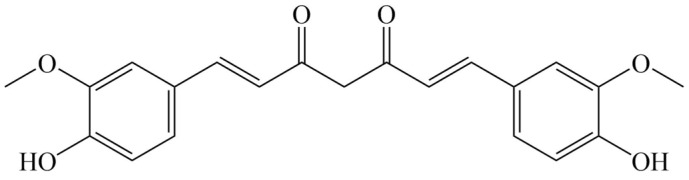
Structural formula of curcumin.

*Curcuma longa* L., a plant belonging to the Zingiberaceae family, thrives in tropical and subtropical regions. It is the primary source of turmeric, a staple in Asian diets and traditional medicine for millennia [1,2]. Its characteristic yellow hue and medicinal properties are attributed to curcuminoids [3], a class of polyketide compounds including the major curcumin, demethoxycurcumin, and bisdemethoxycurcumin [4]. Curcumin (C_21_H_20_O_6_) features a linear heptadiene-dione chain linking two ortho-methoxyphenolic groups (Figure 1), forming an extensive conjugated system responsible for its color and antioxidant activity (via hydrogen atom donation to scavenge free radicals). At room temperature, curcumin is an orange-yellow crystalline powder that is practically insoluble in water but soluble in ethanol, acetone, and chloroform [5].

In traditional medicinal practices, turmeric has long been utilized to enhance blood circulation, alleviate pain, and facilitate liver function [6]. Modern preclinical investigations have further validated its anti-inflammatory, anticancer, antiviral, and neuroprotective properties, thereby fueling growing interest in its potential applications across pharmaceuticals, functional foods, and cosmetics [7,8]. According to recent market research reports [9,10], the global curcumin market was valued at more than USD 628 million in 2024, with an anticipated compound annual growth rate (CAGR) of 11.3% from 2024 to 2030. Currently, North America and Europe dominate the global curcumin market, primarily driven by the demand for high-purity curcumin products and bioavailability-enhanced formulations in dietary supplements and drug development [9]. In contrast, Chinese curcumin market, which reached approximately USD 156 million in 2024, is dominated by industrial-grade products (accounting for over 60% of the market share), largely due to consumers’ price sensitivity, resulting in intense price competition [11].

Despite its promising functional properties, the industrial application of curcumin is hindered by several critical challenges: (1) low extraction yields from conventional natural sources; (2) high cost and environmental impacts associated with chemical synthesis; (3) poor water solubility and low bioavailability, which limit its clinical efficacy; and (4) regulatory hurdles for its application in pharmaceuticals and nutraceuticals [12]. This review synthesizes the latest advances in the production, purification, and application of curcumin, with a focus on systematic quantitative comparison, comprehensive critical evaluation of existing limitations, and actionable strategies for future development.

## 2. Production of Curcumin

Curcumin’s commercial production primarily hinges on three core routes: phytoextraction from the rhizomes of *C. longa* L., chemical synthesis, and microbial biosynthesis. Each route exhibits distinct advantages, limitations, and scalability potential, all of which are quantified and critically compared in the subsequent sections.

## 3. Phytoextraction of Curcumin

**Figure 2:**
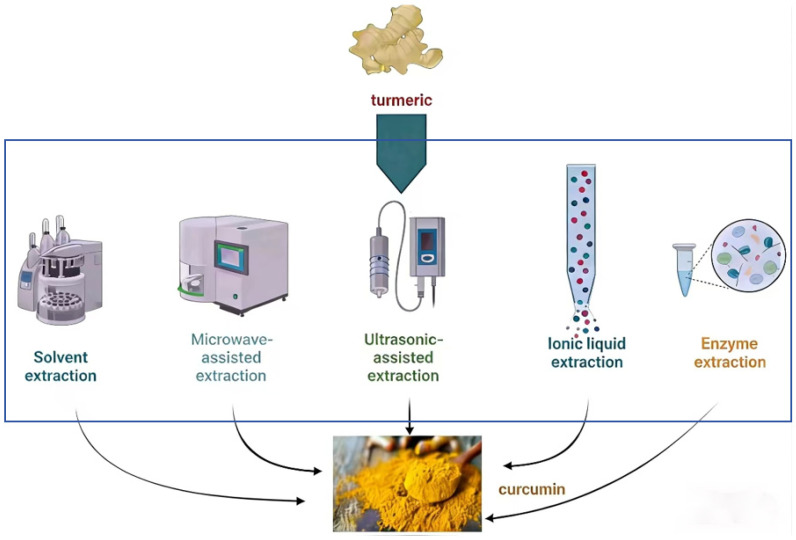
Different extraction methods of curcumin.

Phytoextraction remains the commercial mainstay for curcumin production, as it maintains the natural curcuminoid profile and resonates with consumer preferences for natural ingredients [13]. Its efficiency is contingent upon feedstock quality, extraction techniques, and solvent selection. Traditional solvent extraction methods (maceration, Soxhlet extraction) are simple and cost-effective but are associated with low yields, long extraction times, and degradation of heat-sensitive curcuminoids due to prolonged exposure to heat [14]. Advanced extraction technologies, such as microwave-assisted extraction (MAE) and supercritical fluid extraction (SFE-CO_2_), have been developed to mitigate these limitations, yet their industrial adoption is hindered by high equipment costs, energy consumption, and scalability challenges, particularly for continuous processing (Table 1 and Figure 2). 

### 3.1. Economic and Environmental Trade-Offs of Curcumin Extraction Methods

To conduct a more in-depth investigation into the industrial applicability of these curcumin extraction methods, a comparative analysis of their economic and environmental trade-offs was performed, with key findings summarized as follows:

#### 3.1.1. Cost Breakdown

Traditional solvent extraction requires the lowest initial capital investment, with equipment costs of less than USD 50,000 for small-scale facilities (annual output: 50–100 tons) [22]. However, it incurs higher long-term operational costs, primarily due to substantial solvent consumption (solvent-to-feedstock ratio: 8:1–10:1, *v*/*w*) and additional energy requirements for post-extraction drying processes to remove residual solvents [14]. Conversely, supercritical fluid extraction (SFE-CO_2_) demands a high initial capital investment (USD 200,000–500,000 for industrial-scale units), but its solvent-free nature reduces solvent procurement costs by 60–80% and eliminates the need for drying steps, thereby offsetting partial operational expenses [23].

#### 3.1.2. Environmental Impact

Ionic liquid extraction exhibits a 30–40% lower carbon footprint than conventional solvent extraction, primarily attributed to the low volatility of ionic liquids, which minimizes atmospheric emissions [22]. Nevertheless, the high viscosity of common extraction-grade ionic liquids (200–500 mPa·s at 25 °C, [BMIM][BF_4_]) leads to increased energy consumption for mixing and mass transfer enhancement. Enzyme-assisted extraction minimizes solvent waste, with a low solvent-to-feedstock ratio of 2:1–3:1 (*v*/*w*), but it requires strict temperature and pH control (35–55 °C, pH 4.5–6.0) to maintain enzyme activity; this translates to additional energy input for process regulation (constant-temperature water baths, pH buffering systems) [24]. Life cycle assessment (LCA) further confirms the sustainability gap between traditional and novel extraction technologies: Soxhlet extraction and batch solvent extraction exhibit 58% and 31.2% relative impact in the global warming category, respectively, while ultrasound-assisted three-phase partitioning (UA-TPP) achieves a mere 4.1% relative impact, highlighting the significant potential of process intensification technologies in reducing environmental burdens [25].

#### 3.1.3. Scalability

Traditional solvent extraction and ultrasonic-assisted extraction possess favorable scalability for industrial production, with mature technologies enabling processing capacities of 10–100 tons of turmeric rhizomes per day [26]. In contrast, enzyme-assisted extraction and ionic liquid extraction are currently limited to medium-scale operations (1–5 tons/day). The main bottlenecks include poor enzyme stability during continuous large-scale reactions (leading to reduced yield consistency) and the complex, high-cost recovery of ionic liquids which hinders economic viability in mass production.

#### 3.1.4. Industrial Case Studies

A large-scale turmeric plant in Kerala, India (500 tons/year curcumin) employs a dual extraction mode: solvent extraction for industrial-grade curcumin (food additives, USD 8–10/kg) and SFE-CO_2_ for pharmaceutical-grade (nutraceuticals, USD 30–40/kg). Optimized solvent extraction unit achieves 85% production efficiency (yield vs. theoretical maximum), confirming traditional methods’ large-scale practicality [27].

## 4. Chemical Synthesis of Curcumin

**Figure 3:**
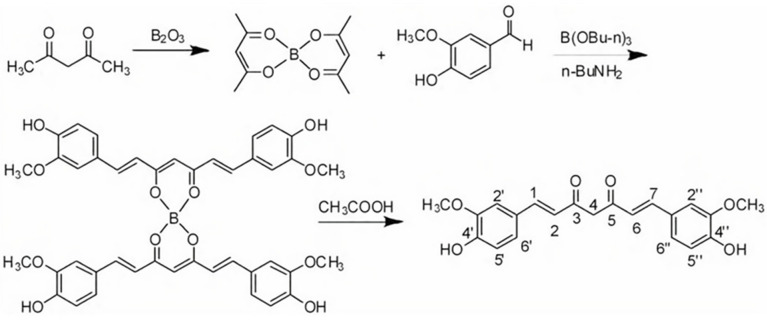
Diacetylacetone boron complex-mediated synthesis of curcumin from long-conjugated chalcones.

Chemical synthesis acts as a supplementary production route to meet increasing market demand, particularly for high-purity curcumin (≥95%) required in pharmaceutical applications [28]. In 1997, the classic synthetic pathway utilized tributyl borate as a catalyst, but was hampered by high costs and flammability risks [29]. However, Yeung et al. optimized this synthetic route using vanillin and acetylacetone as starting materials, thereby reducing costs and enhancing safety profiles (Figure 3) [30]. Building upon this work, Gupta et al. scaled up this method to 100 g batches, achieving a yield of 60% [31]. Subsequently, Nitu’s team further improved efficiency by employing microwave radiation, resulting in a higher yield of ~75% [32].

### 4.1. Quantitative Performance and Limitations

Yield and Purity: Chemical synthesis achieves yields of 50–75% with purity ≥ 95%, which represents a notable advantage over phytoextraction, where industrial-grade products typically exhibit a purity of 85–90% [33]. Cost: The cost of chemically synthesized curcumin is approximately USD 200–300 per kg, offering a considerably lower cost structure compared to curcumin derived from phytoextraction (USD 500–800 per kg) [34]. Environmental and Safety Risks: A significant concern is the utilization of toxic solvents (chloroform, methanol) and the generation of substantial hazardous waste, as indicated by a notably high E-factor (150–200, kg waste/kg product), necessitating strict adherence to environmental regulations [5]. Scalability: While batch synthesis is readily scalable to tonnage levels, continuous production is impeded by the inherent complexity of reaction control and the formation of unwanted byproducts (dicinnamylmethane) [35].

### 4.2. Critical Comparison with Other Routes

Chemical synthesis is cost-competitive for the production of high-purity curcumin; however, it encounters consumer skepticism due to perceptions associated with “synthetic” labeling. Moreover, its environmental footprint, characterized by a high E-factor, is substantially larger than that of microbial biosynthesis [36], thereby limiting its long-term sustainability potential in the food and pharmaceutical industries.

## 5. Microbial Biosynthesis of Curcumin

Enabled via synthetic biology and metabolic engineering approaches, microbial biosynthesis has emerged as a promising sustainable alternative to both phytoextraction and chemical synthesis for curcumin production [37]. By reconfiguring curcumin’s biosynthetic pathway (the phenylpropanoid pathway leading to curcuminoid formation) in microbial hosts, researchers have successfully achieved de novo biosynthesis using renewable carbon sources (e.g., glucose, or glycerol). Key microbial hosts employed for this purpose include *Escherichia coli*, *Saccharomyces cerevisiae*, *Pseudomonas putida*, and *Yarrowia lipolytica* (Table 2).

### 5.1. Biosynthesis Pathways of Curcumin

With the rapid advancement of synthetic biology, the production of natural products through metabolically engineered microorganisms has emerged as a viable and efficient alternative to conventional chemical synthesis. This approach offers notable advantages, including greater environmental sustainability, higher production efficiency, and improved scalability for industrial applications.

**Figure 4:**
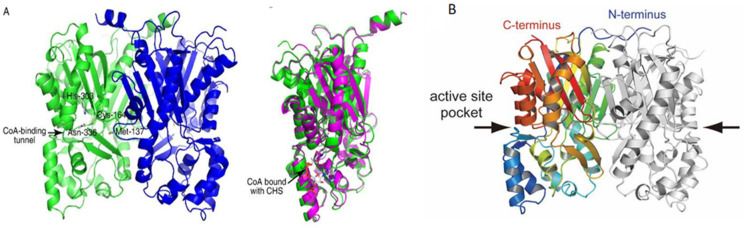
Protein structures of CURS1 and CUS. (**A**) Ribbon diagrams of CHS. Left: Monomer structure, highlighting the CoA-binding tunnel and conserved catalytic residues (His-303, Cys-164, Asn-336, Met-137). Right: CoA-bound CHS complex, with CoA visualized in magenta and the protein backbone in green. (**B**) Ribbon diagram of the CHS homodimer. The C-terminal domain is colored in red/orange, and the N-terminal domain in gray. Arrows indicate the active site pocket in the C-terminal domain, and the interface region of the N-terminal domain.

**Figure 5:**
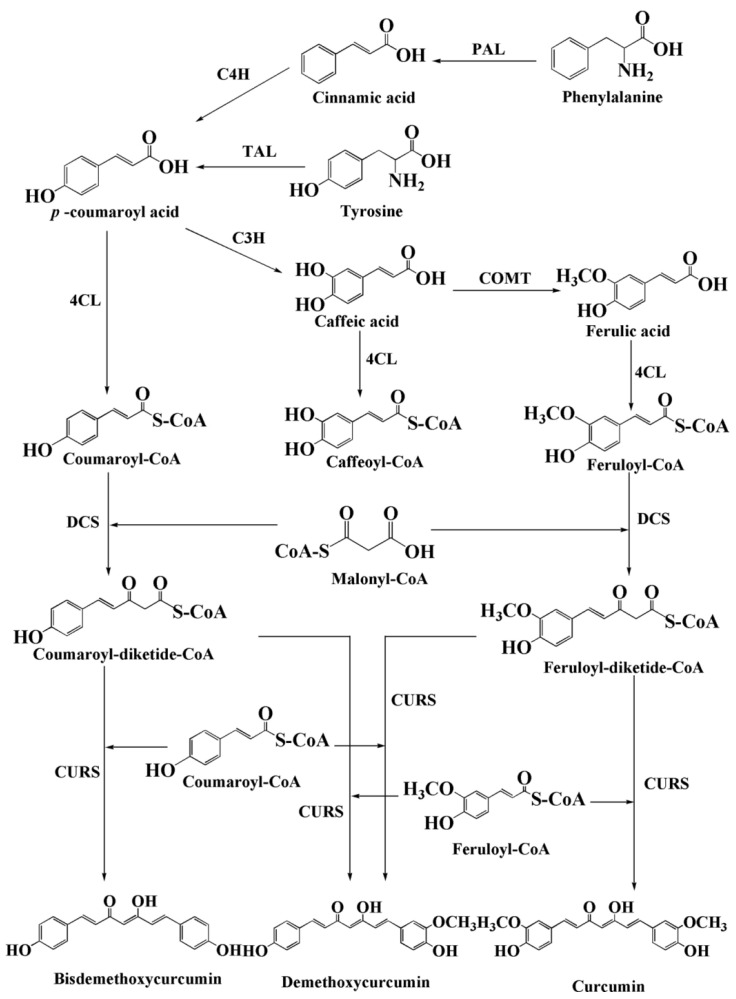
Curcumin biosynthesis pathways. Notes: PAL, Phenylalanine ammonia-lyase; C4H, Cinnamate 4-hydroxylase; TAL, Tyrosine ammonia-lyase; C3H, *p*-coumarate 3-hydroxylase; COMT, Caffeic acid O-methyltransferase; 4CL, 4-coumarate: CoA ligase; DCS, Diketide-CoA synthase; CURS, Curcumin synthase.

Curcumin biosynthesis relies on two key rate-limiting enzymes: diketide-CoA synthase (DCS) and curcumin synthase (CURS) [38]. Katsuyama’s research team [39] successfully determined the three-dimensional structure of CURS1 by means of X-ray crystal diffraction (Figure 4). This enzyme has the characteristics of type III polyketo synthase αββαβα dimer, and the core of its catalytic activity is located in the narrow CoA binding channel. Enzymatic analysis indicates that the catalytic mechanism of CURS1 is significantly different from that of traditional type III polyketo synthase, as it catalyzes the formation of curcumin derivatives through “head-to-head” condensation of polyketone chains [40]. These enzymes sequentially catalyze the condensation of feruloyl-CoA or coumaroyl-CoA) with malonyl-CoA to form curcumin (Figure 5). Heterologous expression of these two enzymes, coupled with optimization of precursor supply pathways has thus been a central focus of metabolic engineering efforts for curcumin microbial synthesis.

### 5.2. Microbial Hosts for Curcumin Production

Various microbial hosts have been engineered for curcumin biosynthesis (Table 2), each with unique advantages, and recent years have witnessed significant progress driven by advanced metabolic engineering strategies.

(1) *E. coli*, a classic model host, has been widely explored via heterologous pathway construction: Katsuyama et al. [41] achieved 113.22 mg/L curcumin by introducing 4CL1, CUS, and ACC with exogenous carboxylic acid feeding, while Chen et al. [42] has advanced *E. coli*-based curcumin production: by developing a multi-level regulation strategy integrating promoter engineering, ribosome binding site (RBS) optimization, and dynamic flux control, the team achieved a curcumin titer of 696.2 mg/L in fed-batch fermentation, representing one of the highest yields reported in *E. coli* systems to date. This strategy effectively enhanced precursor supply and reduced by-product accumulation, providing a valuable engineering framework for industrial-scale production.

Recent advances also encompass novel enzymatic platforms (the iMECS in vitro multi-enzyme system with 126.4 mg/L curcumin and >95% conversion rate [42]), *E. coli* chassis engineering (6.82 mg/L total curcuminoids via tyrosine pathway enhancement and flux redirection [43]), and discovery of novel enzymes (CwPKS1/2 from Curcuma wenyujin enabling direct production of stable curcumin analogues [44]). Collectively, these efforts validate diverse microbial platforms for curcumin production, with advances in enzymatic systems, chassis engineering, and enzyme discovery underscoring a promising landscape for sustainable bioproduction of curcumin and its high-value derivatives

(2) *P. putida*, favored for its diverse carbon metabolism and solvent tolerance, was engineered by Incha’s group [45] to produce dimethoxycurcumin (2.15 mg/L) via activating endogenous synthases and blocking degradation pathways, with co-culture technology proposed as a future direction to boost yields.

(3) The filamentous fungus *A. oryzae* was recently used as a heterologous host to produce curcumin. Curcuminoid synthase (CUS) was expressed in A. oryzae, leading to the synthesis of curcumin when the fungus was cultured with a feruloyl-CoA analog. To enhance curcumin yield, metabolic engineering was employed to increase the supply of malonyl-CoA. This involved two strategies: reinforcing the catalytic activity of acetyl-CoA carboxylase (ACC) to promote malonyl-CoA generation, and disrupting the sterol biosynthesis pathway that consumes acetyl-CoA. The double deletion of SNFA (an SNF1 homolog inhibiting ACC via phosphorylation) and SCAP (a positive regulator of sterol biosynthesis) ultimately resulted in a six-fold increase in curcumin production [46].

(4) *S. cerevisiae* has achieved remarkable progress, including 78.2 mg/L curcumin via pathway optimization [47], 3.5-fold increased malonyl-CoA availability via CRISPR/dCas9 regulation, 152.6 mg/L titers via artificial subcellular compartmentalization, 210 mg/L in fed-batch fermentation with optimized compartmentalization and enzyme fusion [48].

(5) *Y. lipolytica* has gained attention due to its abundant acetyl-CoA pool, with Palmer’s group [49] first demonstrating its feasibility for bisdemethoxycurcumin production (0.17 mg/L) via β-oxidation-mediated acetyl-CoA enhancement.

**Table 2:** Heterologous production of curcuminoids by the microorganism.

Host	Precursor	Key Enzymes or Genes	Yield (mg/L)	Ref.
*Escherichia coli*	Ferulic acid	*At*COMT, *At*4CL, *Cl*DCS/*Cl*CURS; CRISPR/Cas9 editing, pathway optimization.	1400; Batch bioreactor (72 h)	[50]
*Escherichia coli*	Tyrosine	*Fj*TAL, *Pa*HpaB, *Se*HpaC, *Os*COMT, *At*4CL, *Cl*DCS/*Cl*CURS; β-oxidation strategy to enhance Acetyl-CoA.	1000.5; Fed-batch bioreactor (64 h)	[51]
*Escherichia coli*	Tyrosine	*At*PAL, *At*C4H, *At*CPR2, *Fj*TAL, *Pa*HpaB, *Se*HpaC, *Os*COMT, *At*4CL, *Cl*DCS/*Cl*CURS; optimization of fermentation conditions, response surface methodology.	380; Fed-batch bioreactor (32 h) (Modular co-culture)	[52]
*Escherichia coli*	Glycerol	*Fj*TAL, *Pa*HpaB, *Se*HpaC, *At*COMT, *Cl*DCS, *Cl*CURS1, Δ*curA*, Δ*srpB*, Δ*fadR*; Six copy of *At*COMT, *Cl*DCS, Module optimization (TAL/4CL, DCS/CURS).	696.2; Fed-batch bioreactor (64 h)	[42]
*Pseudomonas putida*	*p*-coumaric acid	*Fj*TAL, *At*4CL, *Pa*HpaB, *Se*HpaC, *Os*COMT, *Cl*CUS	2.15; Shake flask (24 h) (YPD)	[43]
*Aspergillus oryzae*	Feruloyl-N- acetylcysteine	*At*4CL/*At*FCL, *Cl*DCS/*Cl*CURS; Curcumin Synthase Overexpression System.	78.2; Fed-batch bioreactor (64 h)	[44]
*Saccharomyces cerevisiae*	Ferulic acid	*At*COMT, *At*4CL, *Cl*DCS/*Cl*CURS; Δ*TRP2*. Co-expression of 4CL1 with *Cl*DCS & *Cl*CURS1.	250; Fed-batch bioreactor (65 h)	[53]
*Saccharomyces cerevisiae*	Glucose	*Fj*TAL, *Pa*HpaB, *Se*HpaC, *At*COMT, *Pp*FerA, *Cl*DCS, *Cl*CURS1; two copy of *Pa*HpaB, *Se*HpaC, *At*COMT.	4,2; Shake flask (72 h) (YPD)	[54]
*Yarrowia lipolytica*	*p*-coumaric acid	*At*COMT, *At*4CL, *Cl*CUS; Δ*POX1-6*, ACC1 overexpression; *At* FAS inhibition.	0.17; Shake flask (48 h)	[49]

Notes: *Arabidopsis thaliana*, *At*; *Flavobacterium johnsoniaeu*, *Fj*; *Curcuma longa, Cl*; *Pseudomonas aeruginosa*, *Pa*; *Salmonella enterica*, *Se*; *Oryza sativa*, *Os*.

### 5.3. Key Advances and Limitations

Highest Titers and Productivity: *E*. *coli* exhibits the highest reported curcumin titer (1400 mg/L) and productivity (19.4 mg·L^−1^·h^−1^) via ferulic acid feeding. However, the high cost of ferulic acid (≈USD 50 per kg) significantly undermines the economic viability of this strategy for industrial-scale applications [55].

De Novo Production from Low-Cost Precursors: De novo curcumin biosynthesis from glucose, a low-cost, renewable carbon source (≈USD 0.5 per kg), has been successfully achieved in both *S*. *cerevisiae* and *E. coli*. Nevertheless, current titers remain relatively low, ranging from 4.2 to 380 mg/L, which limits immediate industrial adoption [45].

Scalability Challenges: Fed-batch bioreactor systems (3–10 L scale) have been validated for *E. coli* and *S. cerevisiae*-based curcumin production. However, scaling up to industrial volumes (1000 L and above) requires further optimization of critical process parameters, including oxygen transfer efficiency, pH homeostasis, and downstream purification workflows [56].

Downstream Cost Burden: Microbial curcumin requires subsequent purification (chromatography, crystallization) to meet pharmaceutical-grade purity standards. Notably, purification processes account for 30–40% of the total production costs, representing a major economic bottleneck for commercialization [57].

## 6. Application of Curcumin

**Figure 6:**
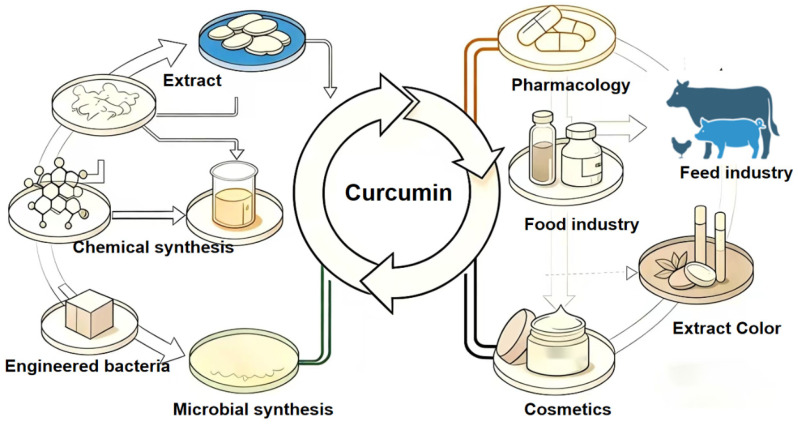
Curcumin application fields.

Curcumin is a natural polyphenolic substance extracted from the turmeric plant. Due to its excellent biological properties such as antioxidation, anti-inflammation, antibacterial and anti-tumor (Table 3), it has attracted much interest. With the continuous advancement of research on it, this compound has shown relatively wide application potential in various industrial sectors. It has application potential in areas such as drug formulations, food additives and animal feed (Figure 6).

### 6.1. Applications of Curcumin in the Food Industry

Curcumin, a naturally occurring yellow polyphenol pigment derived from the rhizomes of *C*. *longa* L., serves as a multifunctional ingredient with significant potential in the food industry. Its primary applications include acting as a natural colorant, preservative, and flavor modifier, leveraging its unique conjugated chemical structure and generally recognized as safe (GRAS) status. As one of the seven globally approved natural pigments, curcumin exhibits exceptional antioxidant capacity, effectively preventing lipid oxidation in meat products and extending shelf life.

In terms of coloring properties, comparative studies by Gomez-Estaca et al. [67] demonstrated that curcumin provides superior color stability and intensity in instant noodles compared to conventional natural pigments like β-carotene and gardenia yellow, making it a preferred choice for enhancing visual appeal in processed foods.

For microbial control, curcumin inhibits the growth of foodborne pathogens such as *E. coli* and Salmonella through multiple mechanisms, including disrupting bacterial cell membrane integrity, inhibiting tubulin polymerization, and inducing oxidative stress. Notably, Zhou et al. [68] reported synergistic antimicrobial effects when curcumin is combined with antibiotics like ampicillin and streptomycin, potentially reducing antibiotic usage in food preservation.

Additionally, curcumin’s distinctive pungent and slightly bitter flavor profile makes it a valuable natural flavor modifier. It is widely incorporated into meat products, noodles, and canned foods to enhance sensory attributes, often complementing other spices and seasonings. However, challenges such as low water solubility and limited bioavailability have prompted research into delivery systems like microencapsulation and nanoemulsions to optimize its application in food matrices.

### 6.2. Application of Curcumin in Feed Industry

The prohibition of antibiotic use in animal feed, first implemented by the European Union (EU) through Regulation (EC) No 1831/2003 in 2006, has since become a global trend, with countries including China issuing similar regulatory measures to address antibiotic residues and antimicrobial resistance (AMR) in livestock products. Against this backdrop, curcumin, as a natural, safe, and multifunctional feed additive, has emerged as a highly valuable alternative to antibiotics, exhibiting substantial application potential in aquaculture, livestock, and poultry production. Accumulating evidence demonstrates that curcumin not only reduces antibiotic dependency but also significantly enhances animal health status and production performance through its antioxidant, anti-inflammatory, and metabolic-regulatory properties.

In ruminant farming, Jaguezeski et al. [69] conducted a controlled trial on lactating ewes and found that dietary curcumin supplementation markedly reduced reactive oxygen species (ROS) levels in peripheral blood, indicating a potent antioxidant effect. Concomitantly, curcumin administration increased ewe milk yield, decreased milk somatic cell count (SCC), and alleviated milk protein oxidation. Notably, SCC is a direct clinical indicator of mastitis (a major inflammatory disease in lactating ruminants), while protein oxidation impairs milk nutritional quality by reducing bioavailable milk proteins. These findings collectively confirm that curcumin exerts dual benefits in improving both the quantity and quality of sheep milk, providing a viable strategy for sustainable ruminant production.

In swine production, curcumin has been proven effective in mitigating metabolic disorders and promoting growth. For intrauterine growth retardation (IUGR) piglets, an issue causing poor growth performance and metabolic dysfunction, curcumin alleviates insulin resistance by regulating the expression of key genes in the hepatic insulin signaling pathway (IRS-1, Akt) and enhancing liver glycogen synthesis, thereby improving nutrient utilization and growth status. Furthermore, Yan et al. [70] demonstrated that supplementing the diet of fattening pigs with 300–400 mg/kg curcumin could completely replace quinolone antibiotics, resulting in a significant increase in average daily gain (ADG) and feed conversion ratio (FCR), as well as improved serum biochemical indicators (reduced pro-inflammatory cytokines and elevated antioxidant enzyme activity), confirming its efficacy as an antibiotic substitute in intensive swine farming.

In poultry production, curcumin-based combinations have shown promise in preventing enteric diseases. Lee et al. [71] reported that the synergistic use of curcumin-enriched turmeric oil and chili oil effectively inhibited necrotizing enterocolitis (NEC), a fatal intestinal disease in broilers, by modulating the mRNA expression of intestinal inflammatory factors (downregulating TNF-α and IL-6, upregulating IL-10) and reducing the production of Clostridium perfringens toxin (the primary pathogenic factor of NEC). This combination strategy not only improves intestinal barrier function but also enhances broiler survival rate and growth performance, addressing a critical challenge in antibiotic-free poultry farming.

Overall, existing research confirms that curcumin supplementation in the diets of livestock and poultry exerts multifaceted benefits: enhancing disease resistance by regulating immune and inflammatory responses, improving nutrient metabolism to promote growth, and elevating the yield and quality of livestock products. To further expand its application in the feed industry, future research should focus on optimizing curcumin delivery systems to improve its stability and bioavailability in feed matrices, as well as exploring dose-effect relationships in different animal species and production stages.

### 6.3. Application of Curcumin in Medical Industry

According to the “Compendium of Materia Medica”, within the framework of traditional Chinese medicine theory, turmeric is regarded as a warm-natured herb. It has a pungent and bitter taste [1]. The pharmacological effects of turmeric mainly lie in promoting the circulation of qi and blood, alleviating dysmenorrhea, relieving pain and reducing swelling, benefiting the liver and gallbladder, and treating rheumatic paralysis. Modern pharmacological research has demonstrated that curcumin, the main active ingredient in turmeric, has great potential in the prevention and treatment of chronic diseases and neurological disorders [72].

Curcumin is a natural substance with both medicinal and culinary value, and it has a very crucial development prospect in the field of medical care. The curcumin-based products currently available mainly highlight their health-promoting functions, such as protecting the liver, supporting bile secretion, and alleviating the symptoms of traumatic arthritis [73]. The formulas of these products mainly include capsules, tablets, oral liquids, and granules, although there are also innovative dosage forms like gummy candies. There is a situation worth mentioning now. The application value of curcumin in the field of tumor prevention and treatment is increasingly attracting the attention of the medical community, and related preparations are gradually entering the clinical application stage. However, it should be particularly noted that the application of curcumin in the fields of medicine and health care products still has limitations at present, such as the relatively single range of product functions [74]. The selection of dosage forms is also relatively limited.

Given such circumstances, future research efforts should focus on expanding the diverse application scenarios of curcumin, developing functional foods for different indications, as well as innovative dosage forms with specific efficacy.

### 6.4. Application of Curcumin in Cosmetics

Curcumin has been employed in the cosmetic industry for several decades due to its antioxidant and anti-inflammatory properties. It has demonstrated potential in a wide range of beauty treatments for the skin, face, hair, lips, and nail care, with beneficial effects against ultraviolet light exposure, aging, inflammation, hair loss, and in the care of lips and nails. Curcumin can be utilized in cosmetics for skin protection, given its properties such as anti-aging, anti-wrinkle, sun-screening, and moisture-retaining capabilities [61]. Curcumin readily degrades when exposed to diverse harsh environmental conditions, including infrared and ultraviolet rays, chemical pollution, and other physical stresses. This degradation can impede the generation of oxygen free radicals and lipid peroxidation, thereby safeguarding the skin against these detrimental environmental factors. Notably, curcumin-loaded formulations can augment its stability and bioavailability to the skin (cellular uptake and penetration behavior) [75]. A continuous supply of curcumin contributes to the enhancement of skin beauty and personal care. Additionally, curcumin is put forward as an anti-bacterial and anti-inflammatory agent for the treatment of acne vulgaris. In a study carried out by Liu and Huang [76], the in vitro skin accumulation and the inhibition of Propionibacterium acnes growth of curcumin-loaded lipid vesicles were assessed. It was discovered that curcumin- loaded lipid vesicles could significantly accumulate in the neonate pig skin and inhibit Propionibacterium acnes. Moreover, curcumin-loaded formulations can effectively penetrate into the hair follicles and can be utilized for the topical delivery of curcumin deep into and through the hair follicles [77]. Potential dermal applications encompass the treatment of acne, inflammation, and hair growth disorders.

### 6.5. Bioavailability Enhancement Strategies: Clinical Evidence and Commercial Formulations

The clinical validation of nano-formulations bridges the gap between curcumin’s biological potential and real-world applications. For the food industry, these formulations enable the development of low-dose, high-efficacy functional foods (curcumin-enriched beverages, baked goods) that deliver meaningful health benefits without compromising taste or stability [78]. For pharmaceuticals, they support the development of curcumin-based drugs for chronic diseases (osteoarthritis, NAFLD) with well-defined pharmacokinetic profiles, a critical requirement for FDA approval [79].

Notably, the success of Theracurmin^®^ and Cavacurmin^®^ demonstrates that bioavailability-enhanced curcumin can achieve commercial viability [80]: Theracurmin^®^ holds a 15% share of the global curcumin supplement market (≈USD 90 million in 2024), while Cavacurmin^®^ is used in over 500 functional food products worldwide [81]. This commercial traction validates the translational value of advanced delivery systems and provides a roadmap for future innovations.

## 7. Conclusions and Perspectives

### 7.1. Limitations of the Study

While this review comprehensively synthesizes the latest progress in curcumin’s production and applications, several limitations should be acknowledged to provide a balanced perspective. First, in terms of literature coverage, we prioritized studies published in the past 5 years to reflect cutting-edge advances, which may have led to the underrepresentation of some classic foundational works that laid the groundwork for current research. Second, quantitative benchmarking across production routes (extraction yields, microbial titers) is inherently subject to variability in experimental conditions (feedstock quality, fermentation parameters) reported in different studies, making it challenging to achieve absolute standardization of comparative data. Third, microbial biosynthesis of curcumin is a rapidly evolving field driven by synthetic biology breakthroughs; despite our efforts to include 2024–2025 studies, some newly reported enzyme engineering strategies or chassis optimization methods may not have been integrated due to the time lag between manuscript preparation and publication. Fourth, while we supplemented regulatory and clinical evidence for curcumin applications, in-depth analysis of long-term clinical outcomes (5-year efficacy data for curcumin-based anti-tumor formulations) and real-world market feedback (consumer acceptance of bioengineered curcumin) remains limited, as such data are scarce in the existing literature. Fifth, the scalability of novel extraction technologies (ionic liquid extraction) and microbial biosynthesis systems has only been validated at pilot scales (1–10 L bioreactors or 1–5 tons/day extraction capacity), and industrial-scale implementation still faces unaddressed challenges (cost reduction in ionic liquids, oxygen transfer efficiency in 1000 L bioreactors) that require further experimental verification.

These limitations highlight critical gaps that future research should address. Specifically, systematic integration of classic and latest studies, standardization of experimental protocols for cross-study comparison, long-term clinical and market validation, and industrial-scale pilot trials will be essential to advance the sustainable commercialization of curcumin. Despite these constraints, this review provides a holistic framework for understanding curcumin’s current status and future directions, with quantitative data and critical evaluations to support decision-making in academic research and industrial applications.

### 7.2. Conclusions

To contextualize these insights within the broader scope of curcumin research, it is important to re-emphasize that curcumin is widely recognized for its prominent anti-inflammatory, antioxidant, and neuroprotective activities, endowing it with broad application prospects in functional food, feed additives, pharmaceuticals, and cosmetics. Despite its substantial biological value, the industrial translation of curcumin remains hindered by three core bottlenecks: (1) low production yields across traditional and emerging routes, leading to high unit costs; (2) poor in vivo bioavailability due to its low water solubility, rapid metabolism, and limited intestinal absorption, which restricts its therapeutic efficacy; and (3) regulatory barriers associated with safety and efficacy validation for high-value applications. Fortunately, recent technological breakthroughs, including advanced green extraction technologies, precision metabolic engineering of microbial cell factories, and targeted nanoformulation strategies, have emerged as viable solutions to address these challenges.

A quantitative comparison of mainstream curcumin production routes further clarifies their respective advantages and limitations: (1) microbial biosynthesis, enabled by synthetic biology, stands out as the most environmentally sustainable route with the lowest E-factor, but its industrial viability is constrained by the need for further yield optimization; (2) phytoextraction, as the primary source of “natural-labeled” curcumin, is favored by consumers and regulatory frameworks for food and cosmetic applications, yet it suffers from poor scalability, relying heavily on *C. longa* L. cultivation cycles, limited raw material supply, and low industrial-grade purity; (3) chemical synthesis offers cost competitiveness for high-purity curcumin but faces intractable environmental risks and consumer skepticism toward “synthetic” labeling in food and pharmaceutical sectors.

### 7.3. Perspectives

To overcome these multifaceted challenges and accelerate curcumin’s commercial translation, future research must prioritize four interrelated directions: (1) quantitative optimization of production routes using green chemistry metrics to balance sustainability, cost, and yield, particularly focusing on engineering high-performance microbial chassis and optimizing fed-batch fermentation processes for industrial scale-up; (2) development of bioavailability-enhanced formulations coupled with rigorous clinical validation to confirm improved in vivo absorption and therapeutic efficacy; (3) resolution of regulatory barriers through large-scale randomized controlled trials (RCTs) to generate robust safety and efficacy data, facilitating the approval of curcumin-based pharmaceuticals and high-value functional foods; (4) adoption of circular economy principles, such as utilizing agricultural by-products as low-cost carbon sources for microbial biosynthesis, and recycling solvents from extraction to minimize waste generation.

By systematically addressing these key challenges, curcumin is poised to transition from a promising natural polyphenol to a leading functional ingredient in high-value products, ultimately catalyzing innovation in the food, pharmaceutical, and cosmetic industries while contributing to improved human and animal health outcomes.

## Figures and Tables

**Table 1:** A summary of extraction methods for curcumin.

Extraction Method	Extraction Rate % (%, Mean ± SD, *n*)	Temperature Range (°C)	Solvent System	E-Factor ^1^	Atom Economy (%)	Advantages	Disadvantages	Ref.
Solvent extraction (maceration)	2.68 ± 0.88, *n* = 5	25–30	Ethanol: water (70:30, *v*/*v*)	120 ± 15	65 ± 5	Simple operation; low equipment cost; controllable conditions	Low yield; long extraction time (24–48 h); heat-sensitive curcumin degradation; difficult solvent removal	[4,13,15]
Microwave-assisted extraction	3.00 ± 0.99, *n* = 6	40–60	Ethanol: water (80:20, *v*/*v*)	85 ± 10	72 ± 4	High yield; short extraction time (10–20 min); low solvent consumption (10–15 mL/g feedstock)	High equipment cost; localized overheating (risk of curcumin degradation); energy-intensive	[16,17]
Ultrasonic-assisted extraction	2.81 ± 0.89, *n* = 7	20–40	Ethanol: water (75:25, *v*/*v*)	78 ± 8	75 ± 3	Low temperature (preserves heat-sensitive curcuminoids); improved yield; simple operation	High equipment cost; low scalability for batch production; uneven sonication	[18]
Enzyme extraction	4.66 ± 1.15, *n* = 4	35–45	Water (pH 5.5–6.0)	62 ± 7	80 ± 4	Highest yield; low solvent use (5–8 mL/g feedstock); mild conditions	Strict enzyme stability requirements (pH, temperature); long reaction time (12–18 h); high enzyme cost	[19]
Ionic liquid extraction	5.72 ± 0.84, *n* = 3	30–50	[BMIM][BF_4_]:water (1:1, *v*/*v*)	45 ± 6	85 ± 3	Green solvent; high stability; short extraction time (30–60 min)	High ionic liquid cost; high viscosity (hinders mass transfer); complex solvent recovery	[20]
Supercritical fluid extraction (SFE-CO_2_)	2.88 ± 0.92, *n* = 5	40–60 (pressure: 30–40 MPa)	CO_2_ + ethanol (5–10% co-solvent)	38 ± 5	90 ± 2	Solvent-free (no residue); high purity; eco-friendly	High equipment cost; limited batch scalability; high energy consumption for pressure maintenance	[21]

^1^ E-factor = mass of waste generated/mass of curcumin produced (lower values indicate higher sustainability).

**Table 3:** Physiological function of curcumin.

Activity	Mechanisms	Associated Diseases	Ref.
Antioxidant	Hinders the production of free radicals	Oxidative stress-related disorders	[58]
Anti-cancer	Induces apoptosis, inhibits metastasis and invasion through multiple molecular targets	Lung, breast, pancreatic, colon, prostate cancers	[59]
Antibacterial	Inhibits foodborne pathogenic and spoilage bacteria	Bacterial infections	[60]
Antiviral.	Inhibits viral gene expression and replication; degrades the ubiquitin-proteasome system	Viral infections	[60]
Anti-inflammatory	Inhibits inflammatory mediators and transcription factors	Arthritis, inflammatory bowel disease	[61]
Trauma healing	Reverses damage to gastric epithelial cells via re-epithelialisation	Gastric injuries	[62]
Antidepressant activity	Increases dopamine levels in the frontal cortex and striatum	Depression	[63]
Antiprotozoal activity	Inhibits thioredoxin reductase and reduces	protozoan proliferation	[64]
Antidiabetic activity	Exerts hypoglycaemic activity	Diabetes	[65]
Anti-AIDS	Inhibits HIV replication and HIV protease activity	HIV/AIDS	[31]
Anti-ischemic activity	Prevents edema and maintains blood–brain barrier integrity	Ischemic injury	[25]
Neuroprotective	Improves memory capacity in Alzheimer’s disease models	Alzheimer’s disease, Neurodegeneration	[66]

## Data Availability

No data was used for the research described in the article.
